# Prevalence and Genetic Variability in Capsid L1 Gene of Rare Human Papillomaviruses (HPV) Found in Cervical Lesions of Women from North-East Brazil

**DOI:** 10.1155/2013/546354

**Published:** 2013-06-20

**Authors:** Ana Pavla Almeida Diniz Gurgel, Bárbara Simas Chagas, Carolina Maria Medeiros do Amaral, Eugênia Maria Bezerra Albuquerque, Ivi Gonçalves Soares Santos Serra, Jacinto da Costa Silva Neto, Maria Tereza Cartaxo Muniz, Antonio Carlos de Freitas

**Affiliations:** ^1^Department of Genetics, Federal University of Pernambuco, Brazil; ^2^Gynaecological Unit, University Hospital Oswaldo Cruz, Brazil; ^3^Gynaecological Unit, Integrated Medicine Center, Brazil; ^4^Department of Histology and Embryology, Federal University of Pernambuco, Brazil; ^5^Molecular Biology Laboratory, Pediatric Oncohematology Center, University of Pernambuco, Brazil; ^6^Cidade Universitária, 50670-901, Recife, PE, Brazil

## Abstract

The aim of this study was to examine the prevalence and genetic variability of the capsid L1 gene of rare HPV genotypes that were found in the cervical lesions of women from North-East Brazil. A total number of 263 patients were included in this study. HPV detection was performed using PCR followed by direct sequencing of MY09/11, as well as type-specific PCR to detect the Alpha-9 species. Epitope prediction was performed to determine whether or not the genetic variants are inserted in B-cell and T-cell epitopes. The prevalence of rare HPV types in cervical lesions was found to be 9.47%. The rare HPV genotypes that were detected were HPV-53, 54, 56, 61, 62, 66, 70, and 81. The genetic variability in the L1 gene of rare HPV types involved thirty nucleotide changes, eight of which were detected for the first time in this study. Moreover, some of these variants are embedded in B-cell or T-cell epitope regions. The results of this research suggest that rare HPV types might be involved in cervical lesions and some of these variants can be found in B-cell and T-cell epitopes. Data on the prevalence and variability of rare HPV types will assist in clarifying the role of these viruses in carcinogenesis.

## 1. Introduction

Cervical cancer is ranked as the third major cause of female cancer worldwide, with an estimate of more than 529,000 new cases diagnosed and 275,000 deaths in 2008 [1]. In developing countries, cervical cancer comprises 85% of the total number of cases [1] and in Brazil, cervical cancer is the third most common cancer among women [2]. 

Persistent infections caused by Human Papillomavirus (HPV) can result in cervical lesions and cervical cancer [3]. HPV is a nonenveloped virus with a circular double-stranded DNA [4]. This virus group belongs to the Papillomaviridae family, which comprises 29 genera and 189 Papillomaviruses (PV) [5]. To date, more than 120 HPV types have been identified and these can be divided into five genera: *Alphapapillomavirus* (Alpha),* Betapapillomavirus* (Beta), *Gammapapillomavirus *(Gamma),* Mupapillomavirus* (Mu), and *Nupapillomaviru*s (Nu) [5, 6]. Among these, 40 HPV types infect the genital tract, 15 of which are considered to be High-Risk (HR) HPV (16, 18, 31, 33, 35, 39, 45, 51, 52, 56, 58, 59, 68, 73, and 82); six species are considered Low-Risk (LR) HPV (6, 11, 42, 44, 51, 81, and 83) and three species are considered Intermediate Risk (IR) HPV (26, 53, 66) [7]. The International Agency for Research on Cancer (IARC) recognizes the HPV-16, 18, 31, 33, 35, 39, 45, 51, 56, 58, 59, and 68 as carcinogenic or probably carcinogenic groups [8]. Moreover, HPV that have a phylogenetic relationship with other carcinogenic HPV are regarded as possibly carcinogenic groups, called Alpha-5, Alpha-6, Alpha-7, Alpha-9, and Alpha-11 species [7].

Recently, epidemiological data on the worldwide incidence of HPV have shown that Alpha-9 and Alpha-7 species cause 91% of invasive cervical cancer [9]. HPV-16, 18, and 45 are responsible for 75% of squamous cell carcinoma and 74% of the adenocarcinoma cases [9]. Despite the high prevalence of Alpha-9 and Alpha-7 species, other Alpha species are involved in cervical cancer, though only to a very limited degree. For instance, in 2010 it was reported that 2.25% of total invasive cervical cancer were caused by rare HPV types [9].

There have been several studies that describe the prevalence and genetic variability of the HPV-16, HPV-18, HPV-31, and HPV-58 genotypes [9–21]. However, few studies have shown the extent of the prevalence and variability of rare HPV types in infected women [9, 16, 22, 23]. Rare HPV types might be involved in cervical cancer due to the genetic variability, especially when these changes are found in linear B-cell or T-cell epitopes, which theoretically allow the escape recognition by the innate immune system. Furthermore, it is still unknown what role they play in the single infections or coinfections that result in cervical lesions and cervical cancer.

Thus, the aim of this study was to investigate the prevalence of rare HPV types, as well as the genetic variability of the capsid L1 gene of these viruses, in cervical lesions of women from North-East Brazil.

## 2. Material and Methods

### 2.1. Population

Cervical cells were collected from November 2010 to August 2011. The samples were obtained from women during their medical consultations at the Gynaecological Unit of the Integrated Medicine Center, in Sergipe State and the Oswaldo Cruz University Hospital, in Pernambuco State, North-East Brazil. A total number of 263 women agreed to participate in the study and signed the consent form. The study included patients with Cervical intraepithelial Neoplasia (CIN) (Grade - I, II, III) and cervical invasive cancer. All the patients were sexually active and their average age was 38.02 (with ages ranging from 16 to 77). This study was approved by the Ethics Committee of the University of Pernambuco (HUOC/PROCAPE 64/2010) and the Ethics Committee of the Federal University of Sergipe (CEP/CCS/UFPE N° 491/11). 

### 2.2. DNA Extraction and HPV Genotyping

The collected cells were placed in polyethylene tubes containing phosphate-buffered saline and then stored at −20°C until analysis. DNA was extracted by using the DNeasy Blood and Tissue Kit 135 (Qiagen) in accordance with the manufacturer's instructions. 

**Figure 1:**
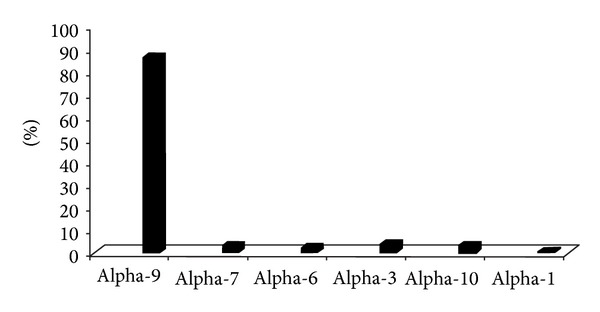
Percentage of *Alphapapillomavirus *species in cervical lesions of women from Pernambuco and Sergipe States, Northeast Brazil. The figure shows the high frequency of A9 species (86.46%), followed by A3 (4.2%), A10 (3.4%), A7 (3.4%), A6 (2.5%), and Alpha-1 (0.8%) HPV genotypes.

HPV DNA detection was performed by using polymerase chain reaction (PCR) with degenerate primers MY09/11 [24], which amplify fragments containing 450 bp from the L1 gene of a wide spectrum of HPV types. In addition, a possible coinfection caused by HPV-16, 31, 33, and HPV-58 was detected through type-specific PCR using primers that target the long control region (LCR), which only allow amplification of the 1000 bp of LCR of each of the above-mentioned HPV genotypes (the primer sequences are described in Table 1) (Figure 1). The reactions were performed with a final volume of 25 *μ*L containing 100 ng of DNA, 1.5 mM of MgCl_2_, 50 *μ*M of each dNTP, 20 pmol of each primer, and 1 unit of Taq DNA Polymerase and 1x buffer. The PCR cycling conditions were as follows: initial denaturation at 95°C for 5 minutes, 35 cycles of denaturation at 95°C for 30 seconds, annealing at 55°C for 1 minute, elongation at 72°C for 2 minutes, and a final extension at 72°C for 10 minutes. The PCR products were run on the agarose gel (1%). 

The positive samples were purified with the Invisorb Fragment Cleanup (Invitek) kit and sequenced (in duplicate) by using ABI PRISM BigDye Terminator Cycle Sequencing v 3.1 Ready Reaction (Applied Biosystems) to obtain both the forward and reverse sequences. 

The sequences obtained were assembled into contigs using the Staden package [25]. They were then evaluated to determine the nucleotide divergence relative to the L1 nucleotide sequences of HPV-53 (NC_001593.1), HPV-54 (NC_001676.1), HPV-56 (X74483.1), HPV-61 (U31793.1), HPV-62 (AY395706), HPV-70 (U21941.1), and HPV-81 (AJ620209). Sequence comparisons were performed using the Basic Local Alignment Search Tool (BLAST) and multiple alignments were carried out by CLUSTALW (Mega 5, Beta version) program [26]. 

New variants were submitted to GeneBank with the following accession numbers: HPV-53-JX912952; HPV-54-JX912948; HPV-56-JX912947; HPV-62-JX912951; HPV-70- JX912950 and HPV-81-JX912949.

### 2.3. B-Cell and T-Cell Epitope Prediction

The putative impact of variability in L1 gene of rare HPV types was estimated in silico by predicting the B-cell and T-cell epitopes. In this study, it was assumed that changes in amino acid sequences of L1 proteins within B-cell and T-cell epitope regions could affect the binding affinities of the neutralizing antibodies and did not initiate an epitope-specific immune response, respectively. Thus, the B-cell epitope of prototype sequences was predicted by using the BcePred server, which is available from URL: http://www.imtech.res.in/raghava/bcepred/. The prediction was carried out with the aid of physico-chemical parameters, such as hydrophilicity, flexibility/mobility, accessibility, polarity, exposed surface, turns, and antigenic propensity [27]. 

The T-cell epitope predictions were performed by using ProPred and ProPred I servers. ProPred I server (available from URL: http://www.imtech.res.in/raghava/propred1/) was used to predict MHC Class-I binding regions [28], while the ProPred server (available from URL: http://www.imtech.res.in/raghava/propred/) was used to predict MHC Class-II binding peptide [29].

## 3. Results

### 3.1. Characteristics of the Population

A total of 263 cervical smear tests were carried out to detect HPV DNA. Of these, 95 cervical smears were submitted to variability studies because they were positive to HPV and also for histopathological findings (CIN I, II, III and invasive cervical cancer). These samples were genotyped and the results showed the presence of HPV-6, 11, 16, 18, 31, 33, 45, and 58 genotypes. With regard to rare HPV types, the following genotypes were detected: HPV-53, 54, 56, 61, 62, 66, 70, and 81 (Table 2). Among these, 9.47% were infected with rare HPV types, while 7.37% of the samples were infected with rare HPV types but without coinfection with other Alpha-9 species.

Twenty-seven cervical samples were coinfected, out of which eleven were infected with the HPV-16/31 genotype, two infected with HPV-16/18, six samples with HPV-31/58, two samples with HPV-16/33, and the remaining samples infected with the following genotypes: HPV-16/58, HPV-16/6, HPV-16/33, HPV-31/33, HPV-16/54, and HPV-16/58/33. 

With regard to the Alpha species, it was found that 86.46% of the positive HPV DNA samples were infected with Alpha-9, followed by Alpha-3 (4.2%), Alpha-7 (3.4%), Alpha-10 (3.4%), Alpha-6 (2.5%), and Alpha-1 (0.8%) species (Figure 1).

### 3.2. Analysis of Variability

 HPV-53 was obtained from patients with HSIL but without coinfection with HPV-16, 18, 31, 33, and 58 types. The analysis of the variability of the L1 gene revealed single nucleotide exchanges in six positions, when compared with the reference sequence. Among these, the mutation point C6942T led to amino acid changes involving P430S in the protein sequence of the L1 (Table 3(a)). Furthermore, the epitope predictions of the HPV-53 L1 protein sequence enabled us to observe that the P430S variation is embedded in the T-cell and B-cell epitope binding regions (Table 3(a)) (Tables 4 and 5).

The patient infected with HPV-54 was coinfected with the HPV-16 type. The L1 genetic variability showed single nucleotide exchanges in five positions when compared with the reference sequence. The A6622T and T6625C mutations have never been described until now. These two mutation points lead to Q313H and S333T amino acid changes in the L1 protein sequence, respectively (Table 3(b)). An analysis of peptide prediction showed that Q313H and T333S variations were found within the T-cell and B-cell epitope binding regions (Table 3(b)) (Tables 4 and 5).

 HPV-81 was detected in one sample with CIN I but without coinfection with HPV-16, 18, 31, 33, and 58 genotypes. An analysis of the genetic variability of L1 showed a new synonymous mutation at the A7148G position (Table 6(a)). The sample infected with HPV-56 was observed without coinfection with other Alpha-9 species (Table 6(b)). Two synonymous mutations were observed in HPV-56 A6666G and A6987G. Moreover, HPV-61 and HPV-66 showed no nucleotide sequence divergence in the L1 gene when compared with the reference sequence.

The analysis of the L1 of HPV-70 was detected in two cervical samples with histopathological findings of adenocarcinoma and CIN I, but without coinfection with HPV-16, 18, 31, 33, and 58 genotypes. The sequence variation of these viruses showed five mutation points, one of which was a nonsynonymous variant. A6886G variation leads to a change in the amino acid (T433A). Furthermore, the T433A mutation is embedded in the T-cell and B-cell epitope binding regions (Table 6(c)) (Tables 4 and 5). 

The genetic variability in L1 protein of HPV-62 showed nine nucleotide exchanges, five of which are nonsynonymous mutations. Among these, two new mutations were found at 6838C and A6921G, which led to E354D and Q382R changes in amino acid, respectively (Table 7). With regard to the altered biological functions, some of these mutation points are included in the T-cell and B-cell epitope binding regions (Table 7) (Tables 4 and 5).

## 4. Discussion

This study described the presence of rare HPV genotypes in CIN I, III and invasive cancer in women from North-East Brazil. The results of this study showed thirty nucleotide changes, nine of which were reported for the first time. Moreover, this study showed the predominance of Alpha-9 and a low frequency of Alpha-7 species in cervical lesions. These correspond to epidemiological data from the rest of the world, which have shown that Alpha-9 is the main species involved in cervical cancer [9]. Furthermore, this study found low frequencies of Alpha-6, Alpha-10, Alpha-3, and Alpha-1 species in cervical lesions or cervical cancer. 

Recently, epidemiological data on the worldwide incidence of HPV have provided evidence that 2.25% of total invasive cervical cancer was caused by rare HPV types [9, 30]. This study showed that 9.47% of cervical samples were infected with rare HPV types, while 7.37% were infected with rare HPV but without coinfection with other Alpha-9 species. Despite the low frequency of these viruses on an individual basis, when all the prevalence data were collated, it was confirmed that rare HPV types are the second most common groups of viruses in cervical lesions of women from North-East Brazil. In addition, recent biological studies have reported that all HPV within Alpha-5, 6, 7, 9, and 11 species contain E6 oncoprotein that degrades p53 [31, 32]. This evidence suggests that rare HPV types play a putative role in carcinogenesis.

The use of PCR with MY09/11 degenerate primers followed by direct sequencing allows other Alpha species to be detected, apart from the Alpha-9 virus group. This study reported rare HPV types, including new variants that were occasionally characterized as single infections. Rare HPV types have also been detected as a single infection in invasive cancer in other studies [9]. 

Some of the rare HPV types found in cervical lesions or cervical cancer in this study are not considered as carcinogenic viruses [8]. Hence, we speculate that the abovementioned genetic variations in rare HPV types might be involved in carcinogenesis due to their higher infection ability. The genetic variations reported in this study could reduce the neutralizing effect of antibodies or make more efficient the interaction between virus and cellular membrane, thus allowing a more effective infection. However, further studies should be performed to demonstrate whether these rare HPV types and their variants can be regarded as carcinogenic. 

In conclusion, this study found a predominance of Alpha-9 species in CIN I, III and invasive cancer in infected women from North-East Brazil. Moreover, genetic variability in L1 genes showed several synonymous and nonsynonymous mutations, which can lead to an altered immune response. Further investigation of the prevalence and variability of rare HPV types can clarify the role of this virus in infections and carcinogenesis.

## Figures and Tables

**Table 1:** Primer sequence used to detect HPV-16, 31, 33, and 58 genotypes.

HPV genotype (LCR)	Sequence primer	Amplicon size (base pairs)
HPV-16	F: 5′-TTCTGCAGACCTAGATCAGTTTC-3′ F: 5′-GTGCATAACTGTGGTAACTTTCTGG-3′	1057 bp
HPV-31	F: 5′-TTAGATCAGTTTCCACTGGGTCG-3′ F: 5′-TTAGTTCATGCAATTTCCGAGGTC-3′	1152 bp
HPV-33	F: 5′-TACCTCCAAAGGAAAAGGAAGACCC-3′ F: 5′-TTGGCACAAATCATGCAATGTTCG-3′	1184 bp
HPV-58	5′ CATGTTCTATGTCCTTGTCAG 3′ 5′ TGA CCC AAA ACG GTT AGT CC 3′	1000 bp

**Table 2:** Rare HPV type found in cervical intraepithelial neoplasia grades I, II, III and invasive cervical cancer.

Rare HPV type	Histopathological grade
HPV-53	CIN III
HPV-54	CIN I
HPV-56	CIN I
HPV-61	CIN II
HPV-62	CIN III
HPV-66	CIN I
HPV-70	CIN I/Adenocarcinoma
HPV-81	CIN I

**Table:** (a)

HPV-53 L1 gene variability	6 6 8 6	6 7 0 6	6 7 4 4	6 9 1 3	6 9 4 7	7 0 0 3	HG
Prototype (NC_001593.1)	C	C	A	G	C	G	CIN III
6 SE	T	T	G	A	T	A	
Protein						P430S	
Hydropathic index						Hydrophobic/Hydrophilic	
Biological functions altered						MHC Class-I/B-cell	

**Table:** (b)

HPV-54 L1 gene variability	6 5 5 2	6 5 7 9	6 6 2 2	6 6 2 4	6 6 2 5	6 9 7 5	HG
Prototype (NC_001676.1)	A	G	A	A	T	C	CIN I
233 PE	T	A	T*	T	C*	T	
Protein	Q313H		**T337S**	**T337S**			
Hydropathic index	Hydrophilic/Hydrophilic		Hydrophilic	Hydrophilic			
Biological functions altered	MHC Class-I/MHC Class-II/B-cell		MHC Class-I/MHC Class-II/B-cell	MHC Class-I/MHC Class-II/B-cell			

**Table 4:** Amino acid residue changes mapped into T-cell epitopes. The amino acids in bold and italic are the positions of change in the predicted T-cell epitopes.

L1 epitopes prediction for T-cell epitopes	Amino acid change insered in epitope sequence
QKDQ***P ***PPEKQDPL	P430S
YWL***Q ***RAQGQNNGI	Q313H
TTRSTNL***T ***LCATAST	T337S
NGICWFN***E ***LFVTVVDTT	E329D
FTICTASTAAA***E ***YKATNFR	A351T, E354D
FDLQFIFQLCKI***Q ***LTPEIMAY	Q328R
HYLQSRAI***T ***CQKGA	T427A
IACQKDAP***T ***PEKKDPY	T433A

**Table 5:** Amino acid residue changes mapped into B-cell epitopes. The amino acids in bold and italic are the positions of change in the predicted B-cell epitopes.

L1 epitopes prediction for B-cell	Amino acid change insered in epitope sequence
TCQKDQ***P ***PPEKQDPLSKYKFWEV	P430S
EYQIFNKPYWL***Q ***RAQGQNNGI	Q313H
VDTTRSTNL***T ***	T337S
N***E ***LFVTVVDTT	E329D
A***E ***YKATNFREFLRHTEEFDLQ	E354D
LQFIFQLCKI***Q ***LTPE	Q382R
CQKDAP***T ***PEKKDPYDDLKF	T433A

**Table:** (a)

HPV-81 L1 gene variability	7 1 4 8	7 2 4 9	HG
Prototype (AJ620209.1)	A	T	CIN I
106 PE	G*	A*	

**Table:** (b)

HPV-56 L1 gene variability	6 6 6 6	6 9 8 7	HG
Prototype (X74483.1)	A	A	CIN I
35 SE	G	G	

**Table:** (c)

HPV-70 L1 gene variability	6 6 3 3	6 7 4 1	6 8 0 1	6 8 7 3	6 8 8 6	HG
Prototype (HPU21941)	G	T	A	A	A	
41 PE	A	·	·	·	·	CIN I
42 PE	A	A	C*	G*	G*	Adenocarcinoma
Protein					T433A
Hydropathic index					Hydrophilic/Hydrophobic
Biological functions altered					MHC Class-I B-cell

**Table 7:** Nucleotide sequence variation in the L1 gene of HPV-62. The vertical numbers indicate the position of the nucleotide variations. HG: Histopathological grade. (*) Substitutions not previously reported.

HPV-62 L1 gene variability	6 7 6 3	6 8 2 7	6 8 3 8	6 8 4 7	6 9 2 1	6 9 2 5	7 0 5 5	7 1 1 4	7 1 5 9	HG
Prototype (AY395706)	A	G	A	T	A	A	A	A	C	CIN III
165 PE	C	A	C*	C	G*	G	G	C	T	
Protein	E329D	A351T	E354D		Q382R		T427A			
Hydropathic index	Hydrophilic/Hydrophobic	Hydrophobic/Hydrophilic	Hydrophobic/Hydrophobic		Hydrophilic/Hydrophilic		Hydrophilic/Hydrophobic			
Biological functions altered	MHC Class-I/MHC Class-II/ B-cell epitopes	MHC Class-I/MHC Class-II	MHC Class-II/B-cell epitopes		MHC Class-I/MHC Class-II/B-cell epitopes		MHC Class-I/MHC Class-II			
